# A Complete Real-World Theory of Language Should Explain How Iconicity Remains a Stable Property of Linguistic Systems

**DOI:** 10.5334/joc.166

**Published:** 2021-08-23

**Authors:** Marcus Perlman, Greg Woodin

**Affiliations:** 1 University of Birmingham, Dept. of English Language & Linguistics, GB

**Keywords:** Language production, Embodied cognition, Semantics

## Abstract

Murgiano et al. make a compelling case for studying iconicity in multimodal face-to-face interaction, but they appear ambivalent about the importance of iconicity at the level of the linguistic system. We argue that, rather than decreasing over time, iconicity is a stable property of languages. Understanding how and why this is so is critical to building a complete real-world theory of language that bridges the situated context of language use with language as an evolving symbolic system. An important point for future research is to examine the interface between iconic prosody and the latent iconic features of words and signs that are frozen in the linguistic system.

## Commentary

Murgiano, Motamedi, and Vigliocco make a compelling case to investigate language within a situated framework, emphasizing the ecological validity of focusing on multimodal communication in face-to-face interaction. They highlight several illuminating studies that support their argument, particularly regarding the vital role of iconicity in early language learning (e.g., Motamedi et al., 2020; Perniss et al., 2018). In their conclusion, the authors argue for a framework that “incorporates the language as a system perspective … into the wider view that language is situated”. However, while stressing the importance of iconicity in face-to-face interaction, they appear ambivalent about its importance at the level of the linguistic system, where they describe iconicity as “subtle”, “underestimated”, and “non-trivial”. Even as the authors summarize research that has established iconicity at the lexical, sub-lexical, and syntactic levels of both spoken and signed languages, their Figure 1 presents speech and sign as distinct from iconic communication. Regarding iconicity in the evolution of languages – while discussing some exceptions, particularly in signed languages – Murgiano et al. appear to largely take for granted the idea that linguistic systems generally show diachronic movement towards arbitrariness.

Here, we argue that it is a misconception that languages generally become more arbitrary over time. Although many words and signs certainly do become less iconic, all evidence suggests that languages do not. Iconicity is prevalent across all known languages, signed and spoken. In spite of Frishberg’s (1975) observation that iconic signs tend to become more arbitrary, relatively mature signed languages continue to feature a high proportion of iconic signs (e.g., Pietrandrea, 2002). Even English, the study of which Murgiano et al. blame for the historical over-emphasis on arbitrariness in linguistics, features iconicity throughout its vocabulary (Winter & Perlman, 2021). Such findings imply that iconicity is a stable property of these linguistic systems, and not just a characteristic of situated communication contexts. Understanding how and why this is so is critical to building a complete real-world theory of language – particularly one that bridges the situated context of language use with language as an evolving symbolic system.

As further evidence for the hypothesis that languages become less iconic over time, Murgiano et al. also cite experiments with Pictionary-style communication games in which drawings of referents such as *Clint Eastwood, cartoon*, and *school bus* became less iconic over multiple rounds (Garrod et al., 2007; Theisen et al., 2010). However, in other experiments using different modalities to communicate about different kinds of referents, iconicity has been found to increase and stabilize over repeated interactions (Little et al., 2017; Erban Johansson et al., 2021). For example, in one study, participants played a 10-round game of charades using non-linguistic vocalizations to express meanings like *rough, slow*, and *big* (Perlman, Dale, et al., 2015). Iconicity was later assessed by testing whether naïve listeners were able to infer the meanings of these vocalizations. The results showed that vocalizations produced toward the middle and end of the game were more iconic than those produced at the beginning. Thus, the vocalizations first became more iconic and then remained that way, even as they exhibited qualities associated with conventionalization, such as becoming briefer in duration, more stable in form, and more formally contrastive with other vocalizations in the system. Communication games therefore do not show an inexorable march towards arbitrariness. Rather, the level of iconicity in a system may stabilize depending on factors such as the modality and semantics of the communicative task. Importantly, in no case to our knowledge has a study shown that iconicity goes away entirely.

The fact that iconicity is a stable property of languages raises the question of how face-to-face interaction drives this stability. One potential explanation is what Flaksman (2017) calls the iconic treadmill, a unidirectional cycle in which new iconic words are coined, lose their iconicity over time, and are then replaced with new iconic coinages. Thus, at the level of interaction, language users would maintain iconicity in the linguistic system primarily by inventing new iconic words or signs – ad-hoc vocalizations or gestures that undergo conventionalization – when one is needed to fill a semantic gap. Yet, while this process may be part of the story, it falls short of integrating iconicity at the system level with the abundance of iconicity featured in situated face-to-face communication, such as the iconic prosody regularly used in speech and sign.

Murgiano et al. note that, in signed languages, iconicity can be “frozen” in lexical signs and then elaborated during language use – an online process that opposes “the system-wide diachronic tendency for forms to become less iconic”. Perhaps similarly, iconic prosody is commonly used to enhance the expressivity of speech (Dingemanse et al., 2016; Perlman, Clark, et al., 2015; Shintel et al., 2006). However, little is known about whether and how iconic prosody in spoken language may grow out of latent iconic features of spoken words, and whether such iconic elaboration may create new, distinct variants or otherwise drive words to become more iconic over time (e.g., the derivation of *teeny* from *tiny* by accentuating the high frequency segment of the diphthong /aɪ/). Thus, there is potential for this process – the activation of frozen iconicity in situated language use that drives words and signs to become more iconic at the system level – to be more common than realized. To the extent that this process happens on a regular basis, it poses a bidirectional account of the balance between iconicity and arbitrariness in linguistic systems.

In conclusion, we emphasize the need to investigate the interface between iconicity in multimodal, face-to-face interaction and the iconic resources of the linguistic system. We believe the points we have made here are largely consistent with the framework advanced by Murgiano et al., and indeed, our perspective has been greatly influenced by the research of the authors and their colleagues. Our goal has been to make explicit certain assumptions and alternative hypotheses about iconicity in languages and how they evolve. In doing so, we hope to have highlighted a key aspect of the situated language framework put forward by Murgiano et al.
